# Young female cancer patients’ experiences with fertility counselling and fertility preservation—a qualitative small-scale study within the Danish health care setting

**DOI:** 10.1080/03009734.2016.1204394

**Published:** 2016-07-13

**Authors:** Didde Hoeg, Lone Schmidt, Kirsten T. Macklon

**Affiliations:** a Department of Public Health, Section of Social Medicine, University of Copenhagen, Denmark;; b The Fertility Clinic, section 4071, University Hospital of Copenhagen, Rigshospitalet, Denmark

**Keywords:** Cancer, fertility preservation, preconceptional counselling, qualitative method, semi-structured interviews, systematic text condensation

## Abstract

**Introduction:**

Fertility counselling for young women newly diagnosed with cancer is an important field of preconceptional counselling. This qualitative, small-scale study explored how young women newly diagnosed with cancer experienced specialized fertility preservation counselling and treatment in the public Danish health care system.

**Methods:**

Semi-structured, in-depth interviews were conducted with five women below 40 years recently diagnosed with cancer. All women received fertility counselling by a fertility specialist at the Fertility Clinic, University Hospital of Copenhagen, Denmark before initiation of cancer treatment. Participants were interviewed at a place chosen by them, and interviews were recorded and transcribed verbatim. Data were analysed using systematic text condensation developed by Malterud and inspired by Giorgi’s phenomenological analysis.

**Results:**

None of the participants were aware that chemotherapy could destroy their eggs. The participants described how specialized fertility counselling and fertility preservation contributed to a belief in life after cancer, which gave them hope that they would survive their cancer disease. Further, the women described how the possibility of fertility preservation removed a huge concern and enabled them to concentrate on their cancer treatment and on getting better.

**Conclusion:**

Overall, the specialized fertility counselling and treatment to preserve fertility was highly valued. The women felt it gave them a choice about their future fertility. The fertility expert presented the various fertility-preserving scenarios, and the women were content that they had an actual choice.

## Introduction

All over the industrialized world, there is a trend among both men and women to delay the birth of a first child (1). Previous qualitative interview studies from the Nordic countries found that both men and women have various priorities when planning their lives, and having children is one of them. However, many had never reflected on their own reproductive capacity; their fertility is often taken for granted (2–4). Unfortunately, some people experience unpredictable events in their lives, which will affect their reproductive capacity. Preconceptional counselling is therefore needed in various special situations adjusted to people’s life situations and special needs. A new and important field regarding preconceptional counselling is the specialized fertility counselling among young women, newly diagnosed with cancer.

In Denmark approximately 900 women below 40 years are annually diagnosed with cancer (5). Over the years cancer treatments have become more effective, allowing more people to survive (6). Therefore, it is important that the health care system focuses on side effects to cancer treatment, such as infertility. Chemo- and radiation therapy may have very detrimental effects on the ovarian reserve, leading to premature ovarian insufficiency (POI) and infertility. In order to circumvent these very unwanted side effects, methods have been developed to preserve the fertility in girls and young women with cancer. Cryopreservation of embryos, oocytes, or ovarian tissue is a technique currently offered to women to preserve fertility (7,8).

Several studies indicate that fertility issues are very important to younger women with cancer (9–15). Studies from Canada, Sweden, and the US showed that 50%–60% of women below 40 years with breast cancer or other types of cancer expressed a wish to have one or more children in the future (12,14,15). Also, a systematic review found that child-bearing after breast cancer is an important issue for survivors (16). Previous studies have shown that fertility counselling by a specialist is particularly important for women newly diagnosed with cancer (9–14,17), and that early referral to a fertility specialist who can provide clear consistent advice can help the women make an informed decision about fertility preservation (13). A Swedish study found that fertility-related communication is not sufficient, and the women in this study called for more individualized information about their fertility after cancer and information about how cryopreservation of embryos, oocytes, or ovarian tissue is performed (18). A quantitative Dutch study argues that more attention should be paid to improve fertility preservation counselling, so that the women understand what fertility preservation involves and feel supported in their decision-making process (19). Additionally, it has been shown among childhood cancer survivors that the risk of infertility affects their well-being in adulthood (20).

Fertility counselling for women with cancer is greatly needed according to earlier studies (9–11,13,14,17,18,21). A newly published review states that limited research has addressed the emotional issues that arise in patients experiencing fertility preservation. Furthermore, in most parts of the world the cost of fertility preservation presents a barrier to treatment, if the patient’s insurance does not cover the treatment (22).

The aim of this qualitative, small-scale study was to explore how young women newly diagnosed with cancer experienced specialized fertility-preserving counselling. The women had received the specialized fertility preservation counselling within a few days or a few weeks after the cancer diagnosis and before chemotherapy treatment began. In Denmark specialized fertility-preserving counselling is offered in a public health care system financed by taxes and without payment by the patients. The programme of ovarian tissue cryopreservation started in the year 1999. Since then, almost 800 patients have had tissue frozen, 53 transplantations to 41 patients have been performed, and so far the programme has resulted in 13 children (23).

## Material and methods

### Participants

The participants were recruited between February and May 2014 at the Fertility Clinic, Rigshospitalet, University Hospital of Copenhagen, Copenhagen, Denmark. Inclusion criteria were: women between 18–39 years of age, who had recently been diagnosed with cancer, and who had recently received fertility counselling. The women should be fluent in Danish and mentally and physically well enough to participate in an interview. Five women were asked to participate in the study, and no one declined. We aimed at recruiting participants with as much variation as possible regarding marital status (married/cohabiting or single), motherhood status (having children or not), and different choices of fertility preservation treatments (cryopreservation of ovarian tissue, vitrification of oocytes, or no fertility preservation treatment).

### Procedures

Prior to the invitation to participate in this study all women had received specialized fertility preservation counselling by a fertility specialist (K.T.M.). All women had received written information about fertility preservation and individualized oral information during the counselling. All women in the study were invited to participate in the study by K.T.M. All interviews were conducted by the first author (D.H.), and interview techniques and difficulties during data collection were discussed with author L.S. D.H. is a Master student in Public Health Science and has a Bachelor degree within qualitative sociology. L.S. is a medical doctor with a PhD in qualitative health research. K.T.M. is a clinical fertility specialist. The interview took place at a location chosen by the participant: three took place in the participant’s own home, one at the participant’s workplace, and one in a café chosen by the participant. Interviews lasted between 45 and 90 min. The interviews were conducted using a semi-structured interview guide focusing on the women’s experiences with fertility counselling and fertility preservation. To gain a better and more personal insight into the field of fertility counselling, D.H. observed a fertility counselling session at the Fertility Clinic, Rigshospitalet before conducting the interviews. Interviews were recorded and transcribed verbatim, and transcripts were re-checked against the recording. Transcripts were anonymized with fictive names. The age of the participants ranged from 25 to 38 years. The participants lived in or near the capital of Copenhagen, and all but one were in a relationship. Three of the participants had one child, while two did not have any children (Table I).

**Table I.** Presentation of study participants with age, number of children, marital status, cancer diagnosis, and selected fertility preservation treatment.

Name in the study	Age/children	Marital status	Diagnosis	Fertility-preserving treatment
Camilla	32 years, one child	Married	Breast cancer	Cryopreservation of ovarian tissue
Sanne	25 years, no children	Cohabiting	Brain tumour	Vitrification of oocytes
Katrine	33 years, one child	Married	Breast cancer	Cryopreservation of ovarian tissue
Sara	29 years, one child	Cohabiting	Breast cancer	None
Lotte	38 years, no children	Single	Breast cancer	Vitrification of oocytes

### Analyses

The interview text was analysed using systematic text condensation (STC) developed by Malterud and inspired by Giorgi’s phenomenological analysis. According to Giorgi the purpose of the phenomenological analysis is to develop knowledge of how the informants experience their world of life in a certain field, by looking for essences and essential characteristics of the phenomena under study (24). Like Giorgi’s method, STC implies analytic reduction with specified shifts between decontextualization and recontextualization (25). Each translated interview was analysed through the following structured process: First, a general impression of data was obtained by reading the transcript and finding preliminary themes associated with the participant’s experiences with fertility counselling and treatment. Then the transcript was read once more with the specific aim to identify meaning units containing information about the research question. The identified meaning units were sorted as thematic code groups across individual participants. The contents of the meaning units were condensed and sorted into subgroups. Finally, the meaning units were decontextualized into a consistent statement regarding the participant’s experience. The results are illustrated with quotes from all participants. Cut-outs are marked as […]. The transcripts were analysed using Nvivo 10.0 software. All authors discussed the interpretations of the findings. We have followed the consolidated criteria for reporting qualitative research (COREQ) (26).

### Ethics

The study followed the codes of ethics from the Helsinki II Declaration. According to Danish legislation, interview studies require no approval from an ethical committee.

## Results

We identified three themes regarding how the participants experienced the specialized fertility preservation counselling: 1) The feeling of being unable to control anything; 2) The importance of creating opportunities; 3) The feeling of having an active choice among the different scenarios.

### The feeling of being unable to control anything

None of the women knew that chemotherapy could destroy their eggs. One woman described how her dream about children was devastated when she was told that she could lose her eggs as a result of the treatment.

Truly, a dream was shattered when they told me that my eggs; they could possibly be affected, and that we might not have any children […]. As soon as she said that I could not have children, I was not sick with cancer anymore […] it was completely gone, completely. I had left that behind me […]. (Sanne)

Katrine described how she felt the cancer as something foreign that took control of her and her life. She felt sorrow in the awareness that she might never be able to have any more children.

It is absolutely horrible. To lose control is, well … Actually, it is a bit strange because you take for granted that you can have children and that is not at all something you can count on. […] but that something, you know, like cancer takes the control from you and controls your life for so long […] that is absolutely horrible. (Katrine)

[…] to be sad that you might lose a child you didn’t have … well, it sounds completely crazy, but that is how you feel. (Katrine)

Lotte described how having children is an epoch-making life event, which the cancer can extinguish. The fertility issue was a huge issue to her when she got her cancer diagnosis. It became secondary when she started her cancer treatment, because of existential crises and fear of death.

[…] Am I even going to survive all this? How can I then think of children. […] it was the fear of death that took up most of the time or all the time at that period. […] It is fine, then they are preserved [the eggs] … but it is not at all certain that I am alive in three years. (Lotte)

Camilla described how she felt overwhelmed with information, so the fertility issue did not differ from the other information. Sara described that the oncologist did not talk much about fertility, when she was diagnosed with cancer, and, like Camilla, she seemed to experience the confrontation with the fertility issue as secondary.

### The importance of creating opportunities

Katrine described how she experienced the fertility-preserving treatment as a positive offer. The fertility preservation was a way to create opportunities, where the cancer had set limits.

I have experienced the fertility issue as something very positive. Everything else about the cancer […] it has been like limitations and the fertility has been—is possibilities […]. All the talk about it is, after all, to create possibilities for me, where all the other stuff about the chemotherapy was a huge limitation in itself. Therefore, I have experienced it as something very, very positive […]. In all the darkness, there has actually been something to light it up. That I think has been extremely positive. (Katrine)

Lotte described how the fertility counselling and treatment contributed to her belief in surviving the cancer.

[…] so they take the eggs out and they do all that and it costs a huge amount of money. So they must believe that I will survive this somehow—well, since they bother to do all this, right? (Lotte)

Camilla described the importance in self-determination and that the fertility preservation treatment minimized her risk of menopause and infertility as side effects. This gave her a feeling of regaining some control over her body, and made her feel that she had the opportunity to attain a normal functional body again when she had recovered from the cancer.

The most important thing is that we decide for ourselves. And then, I think, when you get to the other side have as few implications as possible. To enter menopause prematurely, that is a fairly bothersome implication of having had cancer, so when I hopefully get—or when I am declared well—then we should like to reach some sort of normality that things are as they have always been. (Camilla)

This indicates that the women felt that fertility counselling and treatment created some new opportunities for them, when they felt unable to control anything, because of the cancer diagnosis and risk of infertility as a side effect. Lotte described how the fertility preservation treatment removed a huge concern, which she did not have to think about until after the cancer treatment. One reason given for choosing cryopreservation of ovarian tissue was to lower the risk of early menopause. Also cryopreservation of ovarian tissue made the women feel that they would attain some sort of bodily normality again.

The women’s argument against receiving fertility preservation was that it meant another demand on their body, besides the cancer treatment. Sara described the fertility preservation as a substantial load, which possibly could delay her chemotherapy treatment. Lotte described how it is important to consider whether the wish to have children balances the bodily load of fertility preservation. Contrary to this, Camilla and Katrine described how they experienced the fertility preservation as a minor treatment. Even though Sara rejected fertility preservation she is grateful that she has been made aware of the fertility issue and has been offered advice on possibilities, so that she could make her own decision.

### The feeling of having an active choice among the different scenarios

Even though the women have made different decisions concerning fertility preservation, they all described gratitude for the opportunity to preserve their fertility.

[…] I can say that generally I have been really pleased about it. So I just think that it is important that you […] turn the fertility thing into something positive. Not turn it into something negative and say ‘Oh no, now I can’t have children’ and then focus on that, but instead say ‘oh yes, I might have children after all’ and focus on this and get this perspective on it. (Katrine)

Just the fact that there has been someone who would give me the opportunity to get to the other side and choose for myself, that has meant a lot—that has truly meant a lot. (Sanne)

Well … Actually I think that, as a whole, it was fine—positive, kind, and nice people. (Sara)

The women described it as a good experience and service when the various fertility preservation scenarios were presented to them. They were all pleased and comfortable with the ultrasonography of their ovaries and estimate of their ovarian reserve. They further described the importance of the fertility preservation treatment being their option.

[…] she explained it all well and put up the different scenarios in a very clear way so there was still something to discuss. But it was an active choice you had to make—that I think was very important. (Camilla)

The women’s considerations vary in relation to their decision about receiving fertility preservation. Regardless of their decision, several women implied that they did not know their cancer and treatment status when they had to consider the fertility preservation treatment. This complicated their decision-making process. They had to make a decision about the fertility-preserving treatment before they knew about the cancer treatment.

This analysis showed how the women experienced that the fertility-preserving counselling and treatment gave them the opportunity to regain some of the lost control, by having an active choice among the different scenarios that were listed up for them at the fertility preservation counselling.

## Discussion

In this study, it appears that fertility issues are of great importance for women below 40 years diagnosed with cancer, which is in line with results from international studies (9–14,16–18,21).

Previous studies found that potential loss of fertility could have a profound influence on young women and sometimes be more stressful than the cancer itself (13,18). In this study, one woman described how the fertility issue was so distressing to her that it overshadowed her cancer.

A qualitative study from England (13) showed a trend towards childless women worrying about their fertility. Their concern influenced their decision about cancer treatment. They were more willing to opt out of the recommended cancer treatment. A quantitative study from Canada (12) also found that considerations regarding whether or not to accept fertility preservation were affected neither by the woman’s cancer status nor by whether she already had children. In this small-scale study, we got the impression that women without children were especially concerned about their fertility when they were informed about their cancer diagnosis. Further, the participating women with children described how they would have been more concerned about the fertility issue if they had not previously given birth to a child. However, none of the women refused the fertility preservation treatment just because they had a child. Only one refused fertility preservation in order to avoid another stressful demand on her body. Previous studies have shown that 30%–50% of women decide not to pursue fertility preservation, one reason being that in a majority of countries costs for fertility preservation is covered by the patients themselves. Further, women who refused fertility preservation were less likely to regret their decision if they had had pretreatment counselling with a specialist (22).

A Dutch study found that women affected by cancer welcomed the ability to preserve their fertility despite the cancer diagnosis, and that the women associated the fertility counselling and treatment with positive emotions, such as hope, relief, and a reason to live (9). This agrees very closely with the descriptions found in this study. This study contributes to the existing literature by showing that young women newly diagnosed with cancer experience that specialized fertility preservation counselling and treatment gave them the opportunity to look ahead and imagine a life after cancer. One woman described how the fertility counselling and treatment gave her an indication that the doctors believed she would survive. This indicates that the specialized fertility counselling and treatment contributes to a notion of life after cancer, which gives the women the belief that they will survive the cancer. An American study further found that for younger women the prospect of being able to have children after cancer acts as a powerful stimulus to recover from the disease (27).

Previous studies found that when women received information about possible fertility preservation treatments they felt they were given a choice on this issue and thus on their own future, which was a positive experience (9,18). In the Swedish study, only two women received fertility counselling. The women who did not receive fertility counselling described how they felt they were losing control over their reproductive ability and felt denied the opportunity to participate in decisions concerning future fertility (18).

A study from the UK found that even though women were referred to a fertility specialist, it could be difficult to arrange the counselling because of long waiting times (28). All women in our study told that they were referred to the fertility specialist within a very short time; some of them had to consider the fertility preservation treatment before they knew details about their cancer diagnosis and treatment. This complicated their decision-making process. Still, all women appreciated that they did not experience any waiting time in the fertility preservation process.

Generally, findings in this study thus appear to be consistent with the findings of previous studies in relation to young women with cancer and their experiences with fertility-preserving counselling and treatment.

### Strengths and limitations

In this study it is a strength that all women had specialized counselling with the same professional fertility expert and that the interviewer, through observation, obtained acquaintance with the field before conducting interviews. Further, it is a strength that the participants differed with regard to marital and motherhood status and with regard to which fertility preservation treatment they chose. This we believe has implications on how they experienced the specialized fertility counselling and treatment. All participants were invited by the fertility specialist (K.T.M.) who conducted the fertility preservation counselling. On the one hand this ensured that the women were invited by a health professional they knew beforehand and, further, that women who were very fragile were not invited. On the other hand, this recruitment strategy could imply that the participants felt an obligation to be more positive towards the specialized fertility counselling than they really were. It was emphasized before the interviews were conducted that the participants should feel free to say what they wanted and that participation or not would have no impact regarding their fertility preservation treatment. The main limitation of the study is the inclusion of only five women. The point of saturation during data analyses was not reached. However, the in-depth interviews, lasting 45 to 90 min, contributed with relevant findings to previously published knowledge.

In conclusion, we found that specialized fertility counselling and treatment was highly valued because the women felt they were given a choice about their future fertility. The fertility expert presented the various fertility-preserving scenarios in a satisfying way, and the women were content to have an actual choice. Further, the specialized fertility counselling contributed to a belief in own survival among the women.
